# O007: Lesser and lesser – the impact of small volumes in hand disinfection on quality of hand coverage and antimicrobial efficacy

**DOI:** 10.1186/2047-2994-2-S1-O7

**Published:** 2013-06-20

**Authors:** G Kampf, S Ruselack, S Eggerstedt, N Nowak, M Bashir

**Affiliations:** 1 Bode Science Center, Bode Chemie GmbH, Hamburg, Germany; 2 Institute for Hygiene and Environmental Medicine, Ernst-Moritz-Arndt University, Greifswald, Germany; 3 Development, Bode Chemie GmbH, Hamburg, Germany; 4 Scientific Affairs, Bode Chemie GmbH, Hamburg, Germany; 5 Microbiology, MicroBioTest, Sterling, USA

## Introduction

With some alcohol-based hand rubs a volume of 1.1 mL is recommended per application but it is unknown if such a small volume is sufficient to cover both hands and if it fulfills current efficacy standards.

## Objectives

Aim of our study was to determine hand coverage of three hand rubs (one gel based on 70% ethanol, one gel based on 85% ethanol, one foam based on 70% ethanol) applied with various volumes (all products: 1.1 mL, 2 mL, 2.4 mL, 1 push and 2 pushes; only foam product: 1.1 mL foam, 2 mL foam, 2.4 mL foam).

## Methods

Fifteen subjects applied each product, supplemented with a fluorescent dye with each volume. Quality of coverage was determined under UV light. The efficacy of the three hand rubs was determined according to ASTM E 1174-06 and ASTM E 2755-10. The hands of 12 subjects per experiment were artificially contaminated with *Serratia marcescens* and the products applied as recommended (1.1 mL for the products based on 70% v/v ethanol; 2 mL for the product based on 85% w/w ethanol). The log_10_-reduction was calculated per product.

## Results

A volume < 2 mL yielded a high rate of incomplete coverage (76% - 87%), a volume ≥ 2 mL revealed better results (18% - 40%). There was a significant difference between the five volumes used with all hand rubs (p < 0.001; analysis of variance) but not between the three hand rubs themselves (p = 0.442). Application of 1.1 mL of the hand rubs based on 70% ethanol yielded a log_10_-reduction of 1.85 or 1.60 log_10_ (ASTM E 1174-06) and failed the US FDA efficacy requirement. Application of 2 mL of the hand rub based on 85% ethanol reduced the contamination by 2.06 log_10_ (ASTM E 1174-06) and fulfilled the US FDA efficacy requirement. Similar results were obtained according to ASTM E 2755-10.

## Conclusion

Our data indicate that hand rubs based on 70% ethanol and recommended with a volume of 1.1 mL per application are not suitable to ensure complete coverage of both hands and do not fulfill the current ASTM efficacy standard requirements. Infection control practitioners should try to ensure patient safety by not reducing the volume of hand rub required for adequate for hand disinfection.

## Disclosure of interest

G. Kampf Employee of Bode Chemie GmbH, Hamburg, Germany, S. Ruselack Employee of Bode Chemie GmbH, Hamburg, Germany, S. Eggerstedt Employee of Bode Chemie GmbH, Hamburg, Germany, N. Nowak Employee of Bode Chemie GmbH, Hamburg, Germany, M. Bashir: None declared.

